# Making Use of Comparable Health Data to Improve Quality of Care and Outcomes in Diabetes: The EUBIROD Review of Diabetes Registries and Data Sources in Europe

**DOI:** 10.3389/fcdhc.2021.744516

**Published:** 2021-10-11

**Authors:** Fabrizio Carinci, Iztok Štotl, Scott G. Cunningham, Tamara Poljicanin, Ivan Pristas, Vivie Traynor, George Olympios, Vasos Scoutellas, Joseph Azzopardi, Kris Doggen, János Sandor, Roza Adany, Karianne F. Løvaas, Przemka Jarosz-Chobot, Joanna Polanska, Simion Pruna, Simon de Lusignan, Marcello Monesi, Paolo Di Bartolo, Christa Scheidt-Nave, Christin Heidemann, Inbar Zucker, Anita Maurina, Jana Lepiksone, Peter Rossing, Martti Arffman, Ilmo Keskimäki, Soffia Gudbjornsdottir, Concetta Tania Di Iorio, Elisabeth Dupont, Stella de Sabata, Niek Klazinga, Massimo Massi Benedetti

**Affiliations:** ^1^ Department of Statistical Sciences, University of Bologna, Bologna, Italy; ^2^ Department of Endocrinology, Diabetes and Metabolic Diseases, University Medical Centre Ljubljana, Ljubljana, Slovenia; ^3^ Faculty of Medicine, University of Ljubljana, Ljubljana, Slovenia; ^4^ Department of Population Health and Genomics, University of Dundee, Dundee, United Kingdom; ^5^ Division for Health Informatics and Biostatistics, Croatian Institute of Public Health, Zagreb, Croatia; ^6^ Diabetes Department, Larnaca Hospital Cyprus, Larnaca, Cyprus; ^7^ Health Monitoring Unit, Ministry of Health, Nicosia, Cyprus; ^8^ Department of Medicine, University of Malta, Msida, Malta; ^9^ Health Services Research, Sciensano, Brussels, Belgium; ^10^ Department of Public Health and Epidemiology, School of Health Sciences, University of Debrecen, Debrecen, Hungary; ^11^ Norwegian Diabetes Register for Adults, Norwegian Organisation for Quality Improvement of Laboratory Examinations (Noklus), Haraldsplass Deaconess Hospital, Bergen, Norway; ^12^ Department of Children’s Diabetology, Medical University of Silesia, Katowice, Poland; ^13^ Department of Data Science and Engineering, The Silesian University of Technology, Gliwice, Poland; ^14^ Telemedica Consulting, Bucharest, Romania; ^15^ Nuffield Department of Primary Care Health Sciences, University of Oxford, Oxford, United Kingdom; ^16^ Diabetes Unit “Sant’Anna” Hospital Ferrara, Ferrara, Italy; ^17^ Associazione Medici Diabetologi (AMD), Rome, Italy; ^18^ Azienda Unità Sanitaria Locale (AUSL) Diabetes Unit Romagna, Ravenna, Italy; ^19^ Department of Epidemiology and Health Monitoring, Robert Koch Institute, Berlin, Germany; ^20^ Israel Center for Disease Control, Ministry of Health, Ramat Gan, Israel; ^21^ Sackler Faculty of Medicine, Tel Aviv University, Tel Aviv, Israel; ^22^ Research and Health Statistics Department, Centre for Disease Prevention and Control of Latvia, Riga, Latvia; ^23^ Steno Diabetes Center Copenhagen, Gentofte, Denmark; ^24^ Welfare State Research and Reform Unit, Finnish Institute for Health and Welfare (THL), Helsinki, Finland; ^25^ Faculty of Social Sciences, Tampere University, Tampere, Finland; ^26^ Department of Molecular and Clinical Medicine, University of Gothenburg and Sahlgrenska Hospital, Gothenburg, Sweden; ^27^ Serectrix snc, Pescara, Italy; ^28^ International Diabetes Federation European Region, Brussels, Belgium; ^29^ Department of Public and Occupational Health, Amsterdam UMC, Amsterdam Public Health Research Institute, Amsterdam, Netherlands; ^30^ Hub for International Health Research, Perugia, Italy

**Keywords:** diabetes, diabetes registries, quality of care, performance indicators, risk adjustment, health information

## Abstract

**Background:**

Registries and data sources contain information that can be used on an ongoing basis to improve quality of care and outcomes of people with diabetes. As a specific task of the EU Bridge Health project, we carried out a survey of diabetes-related data sources in Europe.

**Objectives:**

We aimed to report on the organization of different sources of diabetes information, including their governance, information infrastructure and dissemination strategies for quality control, service planning, public health, policy and research.

**Methods:**

Survey using a structured questionnaire to collect targeted data from a network of collaborating institutions managing registries and data sources in 17 countries in the year 2017.

**Results:**

The 18 data sources participating in the study were most frequently academic centres (44.4%), national (72.2%), targeting all types of diabetes (61.1%) covering no more than 10% of the target population (44.4%). Although population-based in over a quarter of cases (27.8%), sources relied predominantly on provider-based datasets (38.5%), fewer using administrative data (16.6%). Data collection was continuous in the majority of cases (61.1%), but 50% could not perform data linkage. Public reports were more frequent (72.2%) as well as quality reports (77.8%), but one third did not provide feedback to policy and only half published ten or more peer reviewed papers during the last 5 years.

**Conclusions:**

The heterogeneous implementation of diabetes registries and data sources hampers the comparability of quality and outcomes across Europe. Best practices exist but need to be shared more effectively to accelerate progress and deliver equitable results for people with diabetes.

## Introduction

The continuous use of comparative health indicators (1) may effectively improve quality of care and outcomes by using targeted strategies including monitoring, benchmarking, audit and feedback (2). Specific experiences show that such approaches work particularly well in diabetes (3), although there is still no general consensus regarding best practices for the design of registries and information systems (4). For this reason, their adoption on a national level still seems quite limited (5).

As a result, many countries use different data sources for the production of indicators at national, regional or provider level (6). This hampers the implementation of common platforms (7) and make international comparisons particularly challenging (8).

To overcome the problem, the European Commission financed specific initiatives addressing the comparability of diabetes indicators across Member States (MS) (9). Between 2004-2017, the EU Directorate of Health and Consumers (DG-SANCO) co-funded three consecutive projects run by the EUBIROD network (10), a coalition of centres maintaining diabetes registries and data sources in 18 countries (11, 12). The aim of the network was to implement common data standards using a federated approach for the systematic production of evidence-based diabetes indicators (11, 13).

In 2012, the network delivered the first fully automated international report using novel distributed analytical software (14). Between 2013-16, the method was further refined as a general platform for the calculation of EU health indicators across all chronic diseases (15).

In 2017, as part of the EU project Bridge Health (16), we carried out a qualitative survey of diabetes registries and data sources in Europe, with the aim of evaluating their organization in terms of coordination, geographical coverage, governance and information infrastructure.

In the present study, we will report the main results emerging from the survey and discuss the availability of comparable diabetes indicators across Europe, on the basis of methodological requirements identified by our previous international projects.

## Materials and Methods

The present study was conducted as part of WP Task 8.2 of Bridge Health (16, 17), a EU co-funded project aimed at defining an overarching framework for the EU Health Information System, run between May 2015 - November 2017. The coordination of this work was facilitated by the Hub for International Health Research (HIRS) and subcontractor Serectrix snc., in collaboration with the EUBIROD network.

The task started with the delivery of a scoping report, presenting the state of the art of 12 collaborating registries and data sources at the first general assembly of the EUBIROD network, held at the University of Surrey, Guildford, UK, 24-25^th^ August 2015. In this session, partners discussed the information collected so far, agreeing to carry out a qualitative survey to collect more structured information on the contents of data sources, through the use of a targeted questionnaire. The details of the instrument were agreed at the first investigators’ meeting, held in Rome on 24-25^th^ November 2015 (http://www.eubirod.eu/projects/bridge/meetings/rome2015/).

The questionnaire was finalised by the coordinating team and shared with all partners before the second meeting. The final structure of the survey questionnaire is shown in (**Table 1**).

**Table 1:** Structure of the survey questionnaire.

***I. Introduction***
Title of the presentation (as specified in the agenda)
Author(s), affiliation (please include full details, address, telephone, email, web)
*Background*
Summary of the activity, characteristics of the registry and role of the organization
*Description of the Activity*
Scope, objectives, main tasks and coverage of the activity in the last 5 years. Structure of the collaboration, centres included in the activity, jurisdictional level, engagement of stakeholders and funding mechanism
***II. Scope of information***
*Research*
Scope and design of research activity, target measurements (clinical parameters, etc), endpoints and analytical models
*Quality monitoring*
Use of the registry for benchmarking and quality improvement in collaboration with relevant stakeholders (e.g. doctors, nurses, patients, etc)
*Policy and Governance*
Use of the results for policy (eg cost assessment/restructuring of services, evaluation of managers, etc)
***III. Technical infrastructure***
*Data systems*
Characteristics of data collection and analysis:
- periodicity (eg continuous live data collection *vs* annual audits, etc)
- type of data (routine administrative, hospital/community/primary care, clinical, EHR, linked)
- geographical coverage (eg regional, national)
- data linkage, privacy and data protection strategies
- details of the data custodian (contacts, etc) and coordinating centre
*IT solutions*
Mechanisms for data collection (eg electronic health records, mHealth solution, etc), data linkage, analysis and reporting mechanisms (eg web applications)
***IV. Outputs***
*Dissemination strategies*
Dissemination and reporting of results (eg public reporting).
*References (last five years)*
*1. Peer review papers*
*2. Quality reports* (eg regional/national benchmarking audits)
*3. Technical reports* (including reports of EU projects)
*4. Press/presentations/videos* (citations of the activity in institutional materials, press articles etc)
*5. Web pages*

The questionnaire included four key sections: 1) description; 2) scope of information; 3) technical infrastructure; and 4) outputs. The description was intended to provide an overview of the organizational aspects related to the registry or data source: affiliation, role, scope, jurisdictional level, stakeholders and funding. The scope of information was investigated using three relevant subsections: a) research items (parameters, endpoint and models); b) quality goals (use for quality and benchmarking); and c) use for policy and governance. The latter was considered as a relevant characteristic of continuous data collection, as the network recognised the importance of institutional involvement for health improvement through targeted initiatives e.g. national diabetes plans. The third area of interest was the technical infrastructure, including all aspects that can determine the capacity of the registries/database to process data and enable international comparisons with the required granularity to allow full automated data exchange. The technical elements also included an examination of potential hurdles in the application of privacy and data protection rules, which were also the subject of an annexed activity of the same task (18). Finally, the questionnaire included a section on outputs, targeting the various dissemination strategies and the key references to scientific papers, technical reports and web outputs produced as part of the routine activity of the participating register/data source during the last 5 years.

The implementation of the survey was carried out by the University of Ljubljana, Slovenia, *via* the open access software infrastructure Redcap (19, 20). The questionnaire was duly implemented to allow the direct contribution of partners *via* a dedicated web page. The survey was finalised between 19^th^ August - 9^th^ September 2017. Results were saved into an Excel sheet and summarized using frequencies and percentages, visualized using pie charts and histograms.

The percent of the total population with diabetes “on record” in the registry or data source during the last year available was calculated using the most relevant estimate of the total population with diabetes available from an official national report or the WHO country profiles (https://www.who.int/diabetes/country-profiles/diabetes_profiles_explanatory_notes.pdf). Such a measure has been used only to provide a figure of the potential contribution that each data source would be able to make for pooled data analysis of distinct subjects with diabetes. Therefore, it may substantially deviate from the coverage of national quality indicators reported from each data source.

Further results included the qualitative assessment of the materials reported in open format by the countries filling the questionnaire. The results contributed to a technical report describing the functioning of the registry/data source in terms of policy, infrastructure, procedures and outputs. The results were discussed among participants at the second investigators’ meeting, held in Nicosia, Cyprus, on 21^st^-22^nd^ September 2017 (see http://www.hirs-research.eu/eubirod/meetings/cyprus2017/). Selected contents contributed to the final report, delivered according to the workplan on 9^th^ October 2017 (15).

In preparation of the present report, further updates were requested directly from all participants, regarding the total number of persons with diabetes included in the data source at the most recent date before 31^st^ May 2021.

## Results

A total of 18 representatives of registries and data sources from 17 countries successfully completed the questionnaire. The full list of institutions, including links to the relevant portals, whenever available, is shown in (**Table 2**). The majority of data sources were located in Northern Europe, with 14 out of 27 EU current MS being represented. Institutions that today are located outside the EU included representatives from the United Kingdom, Norway and Israel.

**Table 2:** Participating countries and coordinating institutions.

Country	Coordinating Institution	Geographical Coverage (name, start year):Number of Centres covered (last yr avail.)	Website (current)
BELGIUM	Sciensano, Belgium	National (2001): 100 Diabetes Clinics	https://www.sciensano.be/en/health-topics/diabetes/role
CROATIA	Croatian National Institute of Public Health	National (CRODIAB, 2000): 2,350 clinics/GPs	https://www.hzjz.hr/sluzba-epidemiologija-prevencija-nezaraznih-bolesti/odjel-za-koordinaciju-i-provodenje-programa-i-projekata-za-prevenciju-kronicnih-nezaraznih-bolest/dijabetes/3/
CYPRUS	Ministry of Health of Cyprus	Regional (2012) 1 hospital 3 primary care centres	
DENMARK	Steno Diabetes Center Copenhagen, University of Copenhagen	National (NDR, 2006)	https://www.rkkp.dk/kvalitetsdatabaser/databaser/dansk-voksen-diabetes-databasen/
FINLAND	Finnish Institute for Health and Welfare (THL)	National (2003, linked data)	https://thl.fi/en/web/thlfi-en/research-and-development/research-and-projects/diabetes-in-finland-findm-
GERMANY	Robert Koch Institute	National (DSS, 2015): DataTrav (2019)	https://diabsurv.rki.de/
HUNGARY	University of Debrecen	National (2016): 20 GPs	http://hmapreg.unideb.hu/
ISRAEL	Ministry of Health of Israel	National (2013), 4 HMOs + Academic	https://www.health.gov.il/UnitsOffice/ICDC/Chronic_Diseases/Diabetes/Pages/Diabetes_registry.aspx
ITALY	Associazione Medici Diabetologi	National (2006); 258 Outpatient Services	https://aemmedi.it/annali-amd/
LATVIA	Centre for Disease Prevention and Control of Latvia	National (1997): 900 GPs/Specialists	
MALTA	University of Malta	Local (1989), Mater Dei Hospital +7 peripheral diabetes clinics	
NORWAY	Noklus	National (2006); 36 hospital outpatient departments + 696 Gps	https://www.noklus.no/norsk-diabetesregister-for-voksne/
POLAND	Medical University of Silesia	Regional (Upper Silesia, 1989): 1 Pediatric Diabetes Clinic	
ROMANIA	Telemedica Consulting	Local (2004), 3 Hospital clinics	
SLOVENIA	University of Ljubljana	National cohort of pediatric population with Type 1 diabetes (1970); Single local adult clinic for all types of diabetes (1982).	
SWEDEN	National Diabetes Register	National (1996)	www.ndr.nu
UK - ENGLAND	University of Surrey	National (RCGP, 1987): 1858 GPs	https://orchid.phc.ox.ac.uk/index.php/rcgp-rsc/
UK - SCOTLAND	University of Dundee	National (SCI-DC, 2002): 40 hospital clinics, 950 GP surgeries	https://www.sci-diabetes.scot.nhs.uk/https://mydiabetesmyway.scot.nhs.uk/

**Figure 1:**
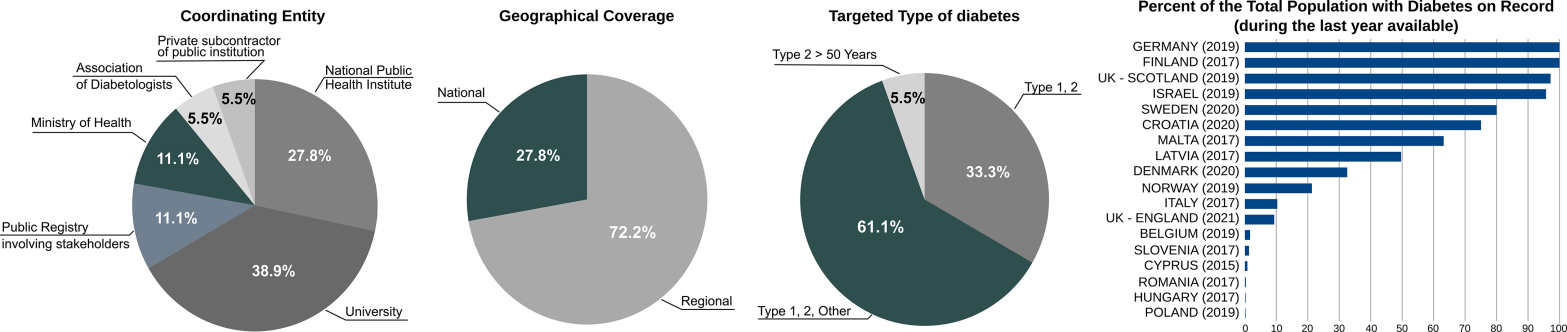
General characteristics of diabetes registries and data sources included in the survey (N=18).

The general characteristics of registries and data sources included in the survey are shown in (**Figure 1**).

The *coordinating entity* was most frequently an academic centre (N=7; 38.9%), followed by a national public health institute (N=5; 27.8%), a public registry involving stakeholders (N=2; 11.1%) or a Ministry of Health (N=2; 11.1%). Only in one case (5.5%), the data source was managed either by an association of diabetologists or a private subcontractor. As described directly by participants, the type of coordination originated from the inspirational guidance of the World Health Organization (WHO) and the International Diabetes Federation (IDF) after the Saint Vincent Declaration in 1989 (21), prompting countries to monitor and control the state of the art of diabetes health care through the use of modern technologies for data collection and exchange (22). The goal has been pursued using different solutions, through the creation of National Diabetes Registries (Scotland, Sweden, Norway, Denmark). In these cases, one central organization (sometimes supported by an academic/research centre) coordinates the activity of different sub-national networks (with the direct participation of multiple stakeholders), or national monitoring is assigned directly to institutional agencies nominated by the Government (Finland, Latvia, Croatia, Germany, Belgium). Less frequently, national activities were carried out directly by the Ministry of Health (Cyprus, Israel). To a lesser extent, medical professional associations and national diabetes audits ensured the activity of diabetes monitoring (England, Italy).

The *geographical coverage* was mostly national (N=13; 72.2%) as opposed to regional (N=5; 27.8%). In general, whenever diabetes monitoring was carried out through governmental action, the geographical coverage was intended to be national, albeit not always the case (see later). The regional emphasis was most often related to academic research conducted on specific cohorts (Poland, Romania, Slovenia, Malta).

All *types of diabetes* were targeted in 11 cases (61.1%), with only Type 1 and/or Type 2 included in additional 6 cases (33.3%). Only in one case (5.5%), the attention was limited to Type 2 over 50 years of age. However, at least in two cases (Slovenia, Poland) the attention to Type 1 was particularly enhanced, as they are typically smaller groups, where the attention to collect detailed clinical data for care optimisation and research is significantly higher.

The *percent of the total population with diabetes at national level* on record in the registries and data sources (based on the last date available) varied considerably across the sample: a total of N=8 databases (44.4%) did not exceed 10%, with additional N=5 (27.8%) achieving between 10-70% and only N=5 (27.8%) over 70%. In some cases, the high percent reflected the method applied for the construction of indicators, particularly with the use of secondary data (Germany, Finland, Israel). The downside of this approach was that clinical indicators were not available for a large population, unless obtained through the use of survey sampling (Germany, Belgium, Hungary). In these cases, the coverage of the population with diabetes could actually be much higher. For example, the sample of 12,000 collected in Belgium reportedly corresponds to 114,200 people with diabetes officially used as denominators for national quality indicators. A high percent was reported by countries with population-based registries in Scotland, Sweden, Denmark, Latvia and Croatia. Notably, professional associations e.g. the Royal College of General Practitioners (RCGP) Research and Surveillance Centre (RSC) in England and the Association of Medical Diabetologists (AMD) in Italy achieved a moderate percentage of people with diabetes, while collecting a large number of relevant clinical parameters.

**Figure 2:**
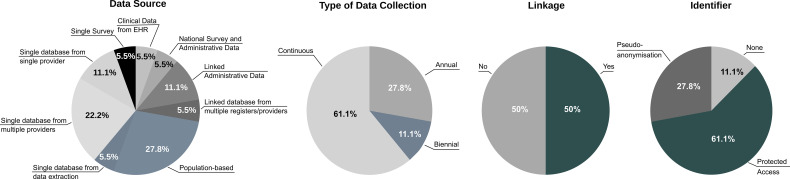
Data characteristics of diabetes registries and data sources included in the survey (N=18).

The data characteristics of registries and data sources investigated in the present study are shown in (**Figure 2**).

The *type of data source* was the most fragmented characteristic in the whole sample. The register was population-based in N=5 cases (27.8%), as opposed to a single database from multiple providers in N=4 cases (22.2%). In N=2 cases each (11.1%), it was either a single database from single provider or composed of linked administrative data. All other cases (5.5% each) where either clinical data from the electronic health record (EHR) linked with samples extracted from national databases, combination of national surveys and administrative data, linked data sources from multiple registries and providers, single database or survey. The different types of data sources used in different countries reflected the existing barriers and enablers present in their evolution. These included a range of different aspects, e.g. limitations of the information infrastructure, varying capacity and skills, lack of interoperability between different levels of the health system, administrative boundaries and heterogeneous implementation of privacy and data protection legislation in each jurisdiction. As a result, with the exception of countries using similar population-based schemes (e.g. Croatia, Denmark, Latvia, Scotland, Sweden), all others sources implemented *ad hoc* solutions.

In the majority of cases, the *type of data collection* was continuous (N=11; 61.1%), as opposed to annual (N=5; 27.8%) or every two years (N=2; 11.1%). Noticeably, the annual data collection seemed to reflect more the periodicity of extraction of the overall linked database, rather than the actual mode of data collection, which in some cases used *ad hoc* software installed in care facilities (Norway, Croatia, Italy). The biennial data collection was a specific characteristic of survey samples (Belgium, Hungary).

The *procedure of data linkage* with other sources was evenly distributed (N=9; 50% either possible or not possible). The extent to which data are not linked across Europe represents a relevant barrier that can limit the information content of diabetes-related databases from a correct epidemiological perspective. This seemed to be directly associated with the type of data source e.g. diabetes clinics (Cyprus, Italy, Malta, Poland, Romania, Slovenia) or general practitioners (Hungary, England). A case in point where administrative and legal issues seemed to be particularly relevant is that of Germany, where different databases were utilised, but could not be linked across.

Finally, a *unique identifier* for patient-based analysis was separately available through protected access in N=11 cases (61.1%), as opposed to being directly accessible in the database *via* a pseudo-anonymised code (N=5; 27.8%) or not available in N=2 cases (11.1%). The latter included two sources using survey sampling for the calculation of indicators (Germany, Hungary).

**Figure 3:**
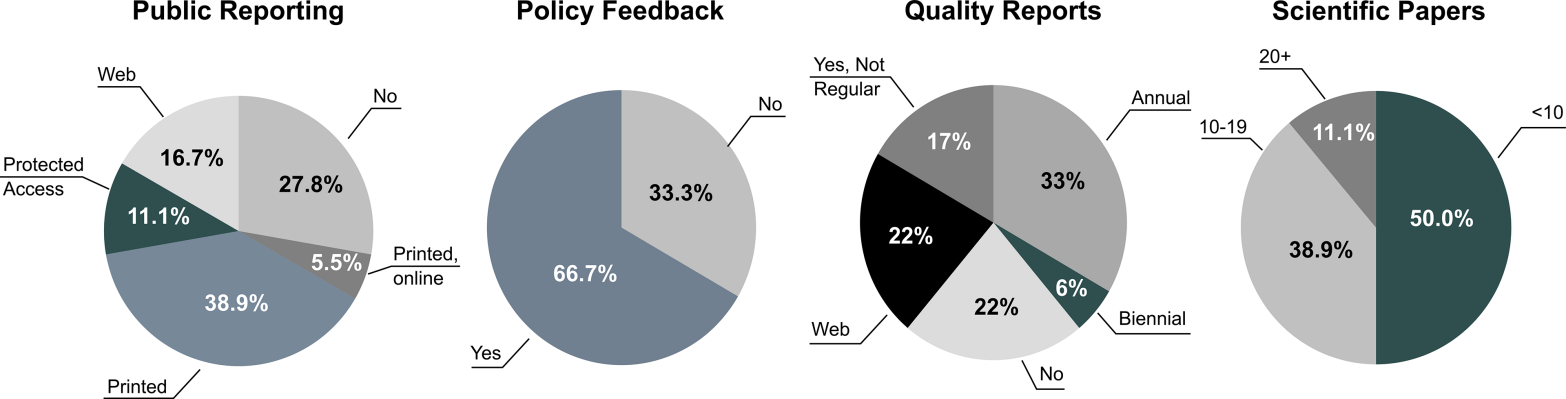
Outputs of diabetes registries and data sources included in the survey (N=18).

A snapshot of the outputs routinely produced by registries and data sources is provided in (**Figure 3**).

Over a quarter of cases (N=5; 27.8%) did not envisage *public reporting*. Among those producing public reports, the majority delivered results only in “printed pdf” format (N=7; 38.9%), one (5.5%) published the same results in printed and online (interactive format), and N=3 (16.7%) as web pages only. The remaining N=2 sources (11.1%) made reports only available through the use of credentials. Different type of institutions did not regularly deliver public reports, including governmental organizations (Cyprus, Finland) and academic/research entities (Hungary, Poland, Romania) which operate for the production of scientific papers. Reports in pdf were more popular among entities delivering annual outputs for audit and performance comparisons. At the other end, national population-based registries seemed to be definitely oriented towards the production of continuous web outputs (e.g. Denmark, Sweden and Scotland).

*Feedback to policy* was foreseen in the majority of cases (N=12; 66.7%), as opposed to none (N=6; 33.3%). A common feature of data sources not using their indicators to provide direct feedback to policy was the lack of a specific agreement between the national/regional authorities to coordinate a formally recognised diabetes register (e.g. Finland, Malta, Poland, Romania, Slovenia, England).

*Quality reports* were available in the majority of cases (N=14; 77.8%), either annually (N=6; 33.3%), biennally (N=1; 5.6%), regularly *via* web reports (N=4; 22.2%) or not regularly (N=3; 16.7%). In N=4 cases (22.2%) quality reports were not produced. A common trait of those using quality reporting was a clear link with non-academic health professionals. In fact, the only data sources that did not appear to deliver quality reports were those using large scale administrative databases or were university driven (Finland, Germany, Latvia and Malta). Nevertheless, quality of care indicators could still appear among those regularly published in public health reports. In the case of Germany, their calculation was possible through a mix of national health surveys and disease management programs (see https://diabsurv.rki.de/Webs/Diabsurv/EN/project/methodology/indicator_set/indicator_set-node.html).

The *scientific production* of peer reviewed publications was unevenly distributed, ranging between 0-90 papers during the last 5 years. In fact, one half of the sample published less than 10 papers (N=9; 50%), the other between 10-19 (N=7; 38.9%) or 20 or more (N=2; 11.1%). The latter included two data sources producing different types of papers: while Sweden (reporting 90 peer reviewed papers) produced articles using large cohort for diabetes epidemiology and quality of care, Poland (reporting 37 papers) worked on clinical parameters involving small groups of individuals with Type 1. The mid group including those who were also productive in terms of scientific literature included large collaborative networks (e.g. Italy and Scotland), national institutes (e.g. Croatia, Denmark, Finland and Germany) and a dedicated research entity (Romania), all being active in diabetes for over a decade. For the majority of data sources, the scientific production appeared to be rather limited.

Further details on the technical infrastructure adopted by data sources included in the present review are included in (**Table 3**).

**Table 3:** Technical Infrastructure of diabetes registries and data sources in the EUBIROD review (Year 2017).

**Belgium (Sciensano)**. A biennial audit collects clinical data from the EHR and demographic data from national databases. A system operates on a single platform for data exchange between health care providers and health authorities, which has been specifically developed by Sciensano. The system consists of data collection software installed locally at each source. The software includes APIs to interface with the local health information system. Alternatively, the data provider can manually fill in questionnaires with/without partial prefilling of available data elements. The data dictionary is managed centrally and made available to data providers. Records are identified by social security number and data are encrypted before transmission to the data landing zone. The latter includes data quality checks and any feedback to the data provider. Data passing quality check are stored in the central data warehouse. A secure web application allows different types of stakeholders to browse reports in the data warehouse.
**Croatia (CroDiab)**. The CroDiab registry is a single data collection system integrating EHR, data from primary care and hospital data as well as administrative and other types of data from different registries (health professionals, population registry…). Data transfer *via* Internet is protected by means of SSL and 128-bit encryption and user authentication. CroDiab provides a complete web-based solution for direct data entry. Data are linked on the basis of a unique personal identifier, then analysed on a person basis for the predefined strata (level of health care, geographical area). County and national level data are presented in crude and standardised format. Web based tools deliver predefined and user customised reports for the population, under user custody. Since 2017, CroDiab has become part of the National Public Health Information System (NAJS), integrated with other components (registries) and external registries (spatial locations registry governed by the State Geodetic Administration, Ministry of Finance PIN registry, etc). Data is provided in an agreed electronic file format, validated and loaded into the system. Authorized users of the National Public Health Information System accessing the CroDiab registry can analyse registry data *via* a web application, through presets linked to a Business Intelligence reporting tool.
**Cyprus (Ministry of Health)**. Clinical and demographic data is collected from medical centres under the coordination of the Department of Information and Technology Services of the Ministry of Health, after every patient visit. All data items are collected using an Access database, specifically built on the basis of the EUBIROD data definitions. The data is primarily recorded in the patient file during the visit, using a template designed to mirror the common dataset. Data are transferred to the electronic registry on a daily basis. Data protection strategies are in place and being adhered to. The Ministry of Health produces annual reports for each data collection center and an aggregate report for all centers, using the specialised BIRO software for clinical audits.
**Denmark (Adult Diabetes Registry)**. Reporting has been automated from EHRs including clinical and demographic parameters collected by general practitioners (GP) and hospitals. Different systems are used in different regions and GP settings. A common platform has been developed to make the different systems interoperable.
**Finland (FinDM – Diabetes in Finland)**. The FinDM Register is gathered by linking data from several national registries using national uniform personal identification codes. The Register collects a broad range of data items that are relevant to the evaluation of diabetes care, with the exception of clinical parameters. The linkages are carried out at THL, the Social Insurance Institution and Statistics Finland using the regular software solutions of these register authorities. At THL, the FinDM Register is in SAS format but for analyses several software packages are used. The current IT solution is related to the status of the FinDM Register as a research database. Recently, researchers have been directed to analyse the FinDM data in the remote access environment Fiona of Statistics Finland.
**Germany (Diabetes Surveillance System)**. Data holders are responsible for storage and data security. Data exchange is only possible by research agreement and under the regulation of data protection and security law. The diabetes surveillance project implemented standard analytical methods to collate information from various data sources, in order to permit continuous disease monitoring and analyses of time trends and regional differences. This served as a basis for sustainable surveillance activities across all non-communicable diseases (NDCs) to assure timely dissemination of results to health policy makers and other stakeholders in the German health care system, for the purpose of evidence-based decision making. All surveys of the health monitoring are designed to be representative for the German population and to monitor the health status, health determinants and health care related aspects in the population. The currently developed and established diabetes surveillance system aims to integrate available secondary data sources in addition to the existing health monitoring system which is largely based on primary health care data collections in national health surveys.
**Hungary (Diabetes National Survey)**. The adjusted version of National Health Insurance Fund T2DM related indicators are evaluated regularly. This system covers the whole country. A survey is conducted in 32 GPs with ethics approval and patients informed consent acquired as a pre-requisite for data collection. The survey includes information on health status, care processes, life style, and socio-economic status. The data quality is regularly checked and corrected according to a written protocol. The data collection and relevant follow-ups for the survey are carried out online. Communication related to quality control is based on email. Performance reports of GP practices are made available online. Each GP can check only own achievement *vs* the national reference.
**Israel (Ministry of Health)**. Data is extracted once a year from the EHR in the four health plans. Data include demographic details and a few clinical parameters. Identifications of the diabetic population is based on the results of blood tests (HbA1c/glucose) performed in the previous year or the purchase of anti-diabetic medications. The database includes individualised de-identified data coded by a coding mechanism that enables cross linking with other databases that are similiarly coded, e.g. the hospitalisation register, mortality register and other national databases (stroke register, blindness register, dialysis register). Data extraction is mandated by national legislation and implemented *via* Excel reports produced by the IT system in each health plan. Sas software is used to analyze the data. Reporting is done electronically through virtual safes.
**Italy (Annali AMD)**. An annual extract is created, based upon the records collected *via* specialised data entry software (sharing a common format), installed at specialist diabetes clinicals adhering to the national network of the Association of Hospital Diabetologists (AMD). A wide set of quality indicators (process, intermediate outcomes, use of drugs, and overall quality of care score) is centrally analyzed. National annual reports on the performance of diabetes clinics, known as “Annali AMD”, are made available online for quality review and benchmarking.
**Latvia (National Diabetes Registry)**. The Register is national, population-based, linked with the Population Register to ensure accuracy of the patients personal data and connected to the Cause of Death Register, to make sure that persons are removed from the active population after death. Data about new cases of diabetes and annual update of information for each patient already in the Register is provided by general practitioners and endocrinologists. A broad range of demographic and clinical characteristics are routinely updated. Information about all registered patients is annually updated by health care professionals using a data entry system called PREDA (Patiens Register Data), a web-based interface using a secure data transmission channel. Only users that are identified, authenticated and authorized have access to PREDA, with access granted only for data entered from that institution. All person-identifiable data are stored encrypted separately from health data, so that not even technicians can identify a single subject. PREDA provides all necessary audit of processes like data writing, editing, reading, etc.
**Malta (Malta Diabetes Data System)**. The database MariaDB developed by the Malta Information Technology Agency (MITA) is installed at the main diabetes clinic at Mater Dei Hospital and at seven peripheral diabetes clinics. The data elements include demographic data, lifestyle characteristics and risk factors, including diabetes specific complications. All doctors working at the diabetes clinics can use the system, although data entry is voluntary. The system was built using the ASP.NET MVC 5 programming model using.Net 4.5 Framework, implemented entirely as a Web API (REST) solution. The Data Layer in the system communicates with the Common Data Repository (CDR). The CDR is a Government web service storing a citizen common dataset used across government departments in Malta. MariaDB is available as a complete day to day Patient Management System and a source of data for reports and research on diabetes.
**Norway (Norwegian Diabetes Register of Adults)**. The registration of data is carried out electronically by general practitioners, endocrinologists, specialists in internal medicine, nurses or other health care workers during regular follow-up appointments. The national register is approved by the Data Inspectorate as a consent-based National Quality Register, thus could be linked to other registries. Demographic and clinical parameters are included in the data registration. Specific computer software has been developed to improve data quality and reduce time required for data entry. Noklus Diabetes is a tool used in hospital outpatient clinics and specialist clinics. Noklus Diabetes Window developed by Medrave AS is a user-friendly tool for data capturing from the various record systems in general practice. Both tools can communicate with the main electronic medical record systems in hospitals and general practice. Noklus Diabetes can also provide decision support and reminders to health care workers. The software is distributed free of charge to participating units. Hospital clinics pay a modest annual license fee to cover the cost of support and development. The register provides annual quality reports to participating centers and individual doctors, comparing results from the local unit against aggregated data from all participating centers. From 2020 the register is no longer consent-based but patients still have the right to opt-out if they don’t want their data to be included in the register.
**Poland (Medical University of Silesia)**. The University has developed its own internet-based database to store and analyse the data on Type 1 patients primarily from the region of Upper Silesia, updated annually. Data are fully anonymized and stored in restricted access area. Demographic, clinical and treatment data are included, with additional information extracted from the NFZ register (medical insurance company), medical services and drug prescriptions.
**Romania (Telemedica Consulting)**. The company supported over the years a number of relevant projects in Romania and the Black Sea region, using data particularly from the RODIAB registry, a system owned by the Ministry of Health, implemented in many diabetes centres in Romania (Bucharest, Cluj, Timisora, Craiova, Iasi, etc.), and TELEDIAB, a diabetes Web-based registry for the Black Sea region, specifically developed by Telemedica Consulting. The database includes demographic and clinical variables and can deliver web-based reports.
**Slovenia (Slovenian National Registry of Childhood Diabetes, Slovenian National Registry of Adult Diabetes)**. The Department of Pediatric Endocrinology, Diabetes and Metabolic Diseases, University Children’s Hospital, University Medical Centre Ljubljana, is responsible for the Slovenian National Registry of Childhood Diabetes. A national cohort of Type 1 diabetes includes complete clinical data from pediatric population that is used for research and quality of care monitoring, in collaboration with the National Institute of Public Health. The Department uses a system implementing openEHR, IHE and HL7. The Department of Endocrinology, Diabetes and Metabolic Diseases, University Medical Centre Ljubljana, is responsible for the Slovenian National Registry of Adult Diabetes and maintains a single local database with clinical data. Open source EDC systems are used to complement the registry database.
**Sweden (National Diabetes Register)**. The Register uses a web-based interface, allowing any relevant caregiver to participate, including primary care and hospital clinics who visit people with Type 1/2 adults and children with diabetes. Data governance within the NHS is strictly aligned to the Caldicott principles. Data are imported from multiple sources and validated based upon clinical templates, allowing to verify that all information according to good quality assurance in diabetes is complete, thereafter exporting data to the Register. In order to create extra value, the web interface and medical records systems offer functions to interact with the individual patient, e.g. reports of all information reported, including medications and risk factors collected during referrals and planned appointments.
**UK - England (National Database of the Royal College of General Practitioners)**. The national network of the Royal College of General Practitioners (RCGP) is coordinated by the Research and Surveillance Centre (RSC) at the University of Surrey (currently at the University of Oxford). Routine primary care EHR data is extracted from the database. The data is collected on a weekly basis and held within a secure server. The data is also pseudonymised, not allowing researchers to identify patients, but still providing a potential linkage with other national datasets. Primary care EHRs are recorded using SNOMED terms and Read codes. The collected data include patient demographics, diagnoses, test results, processes of care, and other important healthcare related data.
**UK - Scotland (Scotland Diabetes Register)**. The Scottish Care Information - Diabetes Collaboration (SCI-DC) system is Scotland’s national suite of Information Technology products designed to underpin Managed Clinical Networks for diabetes. SCI-Diabetes is a national service which utilises real-time data entry and daily batch data import to maintain its shared electronic record. These data include clinical process measures, screenings, long-term outcomes, medication, administrative and correspondence data. Data are linked using the NHS Scotland master patient index (the CHI number) which has been the sole patient identifier in use since 2002. Privacy and protection are covered by UK government policies such as the Data Protection Act and Caldicott principles. NHS Scotland and the University of Dundee are responsible for the ongoing development and maintenance of the SCI-Diabetes systems and its subsidiaries. Data are collected from multiple sources to create an electronic health record for diabetes, covering the whole of NHS Scotland. The data are displayed *via* a web application, available only to users within the NHS intranet who have the responsibility for the care of people with diabetes. Patients can also gain access and contribute to their records e.g. patient-reported outcomes *via* My Diabetes My Way, an online records access website for people with diabetes.
**EUBIROD software (NeuBIRO)**. Members of the EUBIROD network contributed to develop and apply specialised software for automated local reporting, data exchange and international benchmarking. The software was originally conceived in the EU project “Best Information through general outcomes” (BIRO), then successfully tested in the EU project “European Best Information through Regional Outcomes in Diabetes” (EUBIROD) and made generally applicable in the EU project BRIDGE-Health, with the name of NeuBIRO (https://github.com/eubirodnetwork/neubiro). NeuBIRO is an open source, agile, stand-alone multi-platform Java application developed in Groovy (http://groovy.codehaus.org/), using H2 as an embedded DBMS (http://www.h2database.com) and R for all statistical analyses (http://www.R-project.org/). NeuBIRO includes routines to standardise procedures of configuration, data import and quality check, local statistical processing, transfer to central server and central statistical processing. The local and central statistical processing have been integrated into the “statistical engine”, a key component, incorporating procedures for multivariate statistical modelling using micro-aggregates to estimate parameters for risk adjustment *via* multivariate logistic regression across a federated network of health databases.

## Discussion

The results of our survey confirmed the substantial activity carried out in the field of diabetes information in many countries, suggesting three key points for discussion.

Firstly, in terms of main targets of our investigation, our study confirmed that there was not a unique notion of “diabetes registry” (4). A recent review carried out by WHO Europe (23) defined it as “a manually or automatically generated list of people with diabetes, developed as a rule-based system based on specific inclusion criteria”. However, we showed that the same concept has been differently implemented to produce outputs that are similar in their goals of quality of care improvement, surveillance, epidemiology, research, and resource optimisation. Therefore, we prefer to refer for these purposes more generally to “diabetes registries and data sources”.

Secondly, in terms of relevance of information content, we found that these initiatives, even when covering only a portion of the target population or a limited set of indicators, may still be valid for monitoring and benchmarking. Our survey included sub-national data sources which involve a range of stakeholders, in many cases including professional networks that collect clinical parameters otherwise not available through registries relying mainly upon administrative data. These attractive features called for more cohesive initiatives, based on mutual respect and joint commitment between coordinating institutions and physician networks.

Thirdly, in terms of methodology for evaluation, given the complexity and rapid evolution of modern information systems, we demonstrated the advantages of requesting details directly from managers, developers and data custodians of diabetes registries and data sources, who are knowledgeable about the actual operating conditions.

Using this approach, our survey confirmed the activity of a number of mature, well organized national networks that are capable of delivering regular reports on the majority of people with diabetes in their own settings and jurisdictions. Seven out of eighteen entities presented in this report were also included in a recent review of national diabetes registries (5), showing that the measurable results of these initiatives were mild in terms of processes and information gain and very limited in terms of clinical outcomes. Our survey highlighted the challenges faced by national registries designed to respond to different stakeholders, including people with diabetes, policy-makers, health care decision makers, health professionals, research institutions and the general population. The lack of focus on relevant complications (24), as opposed to the production of batteries of statistical indicators, may explain the difficulty in achieving selected clinical targets.

Our study also showed large variability in terms of governance, information infrastructure and generated outputs. The results were obtained through the use of a structured questionnaire, consistently with our driving principles of direct inquiry. The limited production of scientific outputs supports the hypothesis that any assessment based on the systematic review of available outputs would likely introduce bias and miss out on many details of interest.

The scoping review recently carried out by WHO Europe (23) attempted to resolve these limitations using a three-pronged strategy, consisting of: a) search of different types of materials; b) consideration of sub-national registries; and c) open consultation on preliminary findings. However, results are difficult to compare, due to the limited overlap with our sample, possibly owing to restrictive inclusion criteria adopted by the WHO e.g. specific definitions of diabetes registries and information systems, search keywords not including “data source” or “database”, and papers only limited to open access journals.

**Figure 4:**
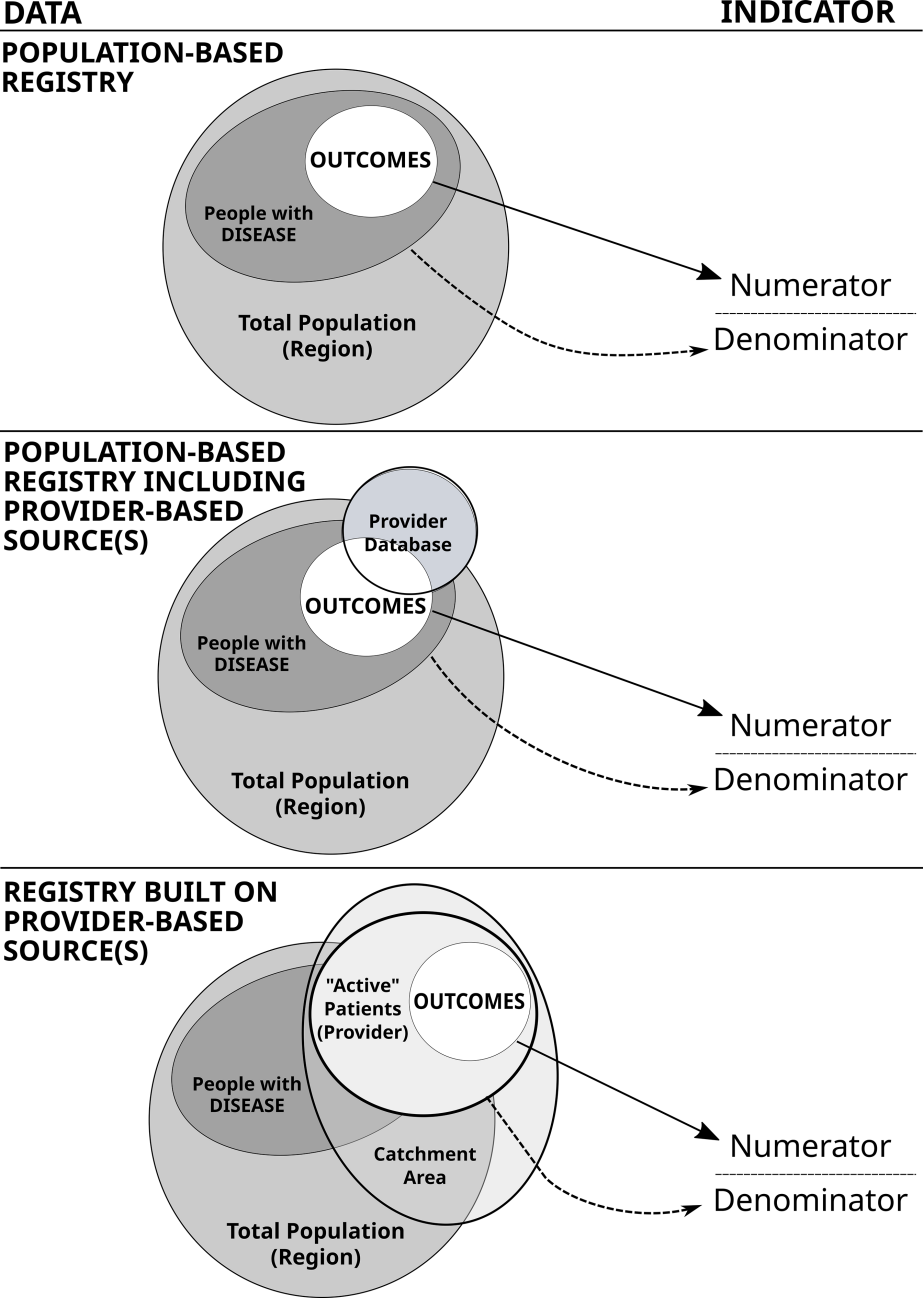
Reliability of indicators derived from different data schemes. At left: different combinations of data sets. At right: the resulting indicator, presented for convenience as the typical quality of care indicator, including the number of cases with negative outcomes in the numerator (e.g. the number of subjects with major amputations) and the reference population in the denominator (e.g. the number of people with diabetes).

In our survey, we found that the *type of data source* was the most variable characteristic among those investigated. This can have critical implications on the reliability and comparability of indicators, deserving to be explained in detail using the models presented in (**Figure 4**).

The first model represents the ideal setting of a “population-based diabetes registry”. Under these circumstances, the number of outcomes occurring in a specific target population (e.g. any/specific type of diabetes, with/without complication, etc) can directly contribute to a person-based numerator, including a fraction of those belonging to a well-defined total population (e.g. population living in a catchment area, local health authority, region or country). This condition is fully met in national registries that also include clinical parameters e.g. Croatia (25), Denmark (26), Latvia (27), Scotland (28) and Sweden (22).

The second model envisages the inclusion of one or more provider-based sources, within or in addition to an underlying population-based registry (or other type of source directly linked to a specific population e.g. administrative data). In this case, a more complex infrastructure is progressively built to increase the completeness of data collection, for example to incorporate new items e.g. clinical parameters that are difficult to collect as the first instance. Under this scheme, a critical problem is to use only those records in the provider database that match the specifications of the population-based registry in both the numerator and denominator. This is possible through the use of a validated unique ID, which we only found in 50% of cases. A case in point related to the national surveillance is that of Germany (29), in which a variety of data sources have been used, but indicators may be generally difficult to report consistently at population level, due to the impossibility to use a common unique id. We have found this to be structurally achieved in the cases of Finland and Israel.

In the third model, indicators are derived only from one or more provider-based data sources. In this case, the percent of the total population on record can be patchy and prone to bias both in the numerators and denominators (unless a unique ID is thoroughly applied, see above). This is the case in which results may be seriously undermined by opportunistic selection (e.g. dropping high risk patients), incorrect identification of person-based profiles (e.g. double counts and difficulty using repeated measures), or uncertain attribution of indicators to a specified reference population or geographical area. In such situations, a list of “active patients” should be maintained to ensure that denominators are correct. As an exemplary case, one could consider the indicator of the number of subjects with HbA1c test done annually. A correct calculation would require a list of those expected to visit the clinic during the year, rather than those actually visiting it. By discarding those unable to visit, the indicator would automatically ignore high risk patients (e.g. those visually impaired), so that all results would inevitably fall in the surrounding of 100% (the main reason for visiting the diabetes clinic being the annual examination). In our survey, given that we could not find explicit mentioning of such a technical aspect, we could argue that this is a specific area worth further investigation. This model is particularly relevant, as it represents the majority of diabetes registries and data sources included in our study.

The taxonomy described above may serve as general guidance, but various conditions may hold under different circumstances that can rapidly change over time, implying the possibility to switch from one model to another. Nevertheless, it is important to note that the interpretation of statistical indicators cannot ignore the underlying information infrastructure, particularly when person-based analysis is required to evaluate the performance of health services.

A characteristic that was also found to be rather variable in our sample was the *coordinating entity*. We found that a small fraction of national registries, namely in Denmark (26) and Sweden (22), were organized as a structural collaboration incorporating multiple stakeholders. This type of agreement may have certain advantages, e.g. the high level of standardization of internal procedures allowing to cover a large portion of the target population, facilitated access to a unique ID through opt in-opt procedures and more transparent rules for health data governance. Although coordinated by academic or professional entities, similar conditions are also present in the Scotland Diabetes Registry (28), the RCGP RSC collaboration in England (30) and the AMD database in Italy (31). Alternatively, the highly structured decision making process found in collegial arrangements can make activities, such as bespoke research and developing new analytical models less flexible. The centrality of public entities in certain cases enhanced internal flexibility, conditional on the availability of resources. The availability of complete electronic health records at medical practices, together with the resolution of all regulatory aspects related to data protection, can harness public health and health care services research in more complex frameworks e.g. Germany (29). In this case, using different sources at a national level has been particularly challenging, but the attention towards collaborative environments has grown out of more targeted surveillance activities. In other cases, universities maintain sources in accordance with contractual obligations and/or to continue their dedicated research activity.

The *geographical coverage* was in almost three quarter of the cases regional, reflecting the diverse characteristics of partners included in the EUBIROD network. Both Type 1 and Type 2 were targeted in almost all cases, with nearly half of the sample covering at least 50% of people with diabetes. *Periodic data collection* was carried out in over one third of cases, with two thirds ensuring continuous updating of their database. There seemed to be no pattern for this condition, except for policy feedback that was consistently ensured in all cases with annual updates.

On the other hand, the characteristic that was more evenly distributed among participants was the possibility of performing *data linkage*, which was possible for half of the cases. This confirms the heterogeneous implementation of principles of privacy and data protection in many EU/OECD countries, despite relevant attempts to harmonise approaches (18). However, we noticed that the availability of a *unique identifier*, either through pseudo-anonymisation or protected access, was indeed possible in nearly 90% of cases. This seems to indicate that the priority given to the uptake of security procedures has not hampered the secondary use of health data (32).

We were able to assess the relevance of a unique ID within the existing technical infrastructure described by participants in the survey. There was a clear role of the master patient index at centres operating in Sweden (22), Croatia (25), Denmark (26), Finland (33), Israel (34), Latvia (27), Norway (35) and Scotland (28). Among them, only Finland was unable to incorporate clinical parameters from medical records, due to the decentralised operations and frequent use of unstructured format. However, the situation is improving and clinical data should be rapidly made available (32). On the other hand, electronic health records collected at the point of care using a valid patient ID were noted in a second group of participants from Belgium (36), Cyprus (37), England (30), Italy (31), Malta (38), Poland (39), Romania (40) and Slovenia (41). For England, the advantages offered by an expanding network of general practitioners included the possibility of linking and comparing items from different comorbid conditions stored in the same database (42). The solution resembles the model of quality registries widely implemented in Nordic countries across different silos (43), with the further advantage of being able to directly access all data in one location. A third group of participants included Hungary (44), where clinical data from the survey were designed to be linked across, and the national surveillance system from Germany (29), in which multiple datasets contributed to different indicators, but could not be linked across using a unique ID.

We found that the information infrastructure determined the type of results that could be delivered by the existing diabetes registries and data sources. Consistently with differences among systems in place, the types of *outputs* were also quite variable. Static formats e.g. pdf documents appeared to be still very popular, while web formats represented almost a quarter of cases. However, an equal fraction of cases did not present results in any public reporting. Interestingly, no specific characteristic seemed to be associated with either approach. On the other hand, *policy feedback* was not provided by one third of participants who seemed more oriented towards research. This may indicate a potential gap between research and policy that should be overcome to fully exploit the availability of actionable information. *Quality reports* were not foreseen by participants who had in common the lack of clinical parameters in the data source and feedback to policy makers. These characteristics confirmed the gap between the availability of health indicators and their direct use in the interest people with diabetes.

In summary, our survey filled the gap between the details available from the literature and the actual operating characteristics on the ground. Previous results from the EUBIROD network showed that the majority of parameters included in the International Consortium for Health Outcomes Measurements (ICHOM) standard set (45) were already present in many sources but presented varying quality and completeness (13). In this study, we showed that the general features, data characteristics and outputs delivered by systems in place may explain much of the variation found before. Although best practices exist, the ecosystem of diabetes information systems appears still fragmented and not immediately related to the expectations of all primary stakeholders.

These problems can be resolved by strengthening international collaboration through a federation of the existing initiatives. Building a European Diabetes Registry could be a convenient strategy to prepare health systems for future emergencies (46), while fulfilling the goal of the EU Parliament “*to coordinate, collect, register, monitor and manage comprehensive epidemiological data on diabetes, and economic data on the direct and indirect costs of diabetes prevention and management*” (47).

Furthermore, combining multiple large databases can provide essential data for training, testing and validating meaningful predictive models that would enhance strategies for the reduction of diabetes complications. For example, it would be possible to move from monitoring traditional clinical parameters e.g. hypertension (39) to more complex investigation carried out in real time e.g. amputation free survival (48). In this way, people with a moderate complication e.g. diabetic foot ulcers may be systematically followed up using information from their baseline profile (e.g. type and duration of diabetes), in association with clinical parameters (e.g. HbA1c), other diabetes complications (e.g. diabetic retinopathy) and detailed morphological characteristics of foot ulcer (using medical imaging). The infrastructure can be rapidly scaled up to incorporate innovative approaches that are revolutionising the way population-based registers are linked to personal data e.g. those captured *via* smartphone apps (49). The possibility to provide access to micro-aggregate data extracted from such common data models across Europe would represent an ideal case study for the implementation of the European Health Data Space (50). This is an intrinsic functionality of the software implemented by the EUBIROD network at its inception (15), which respects the principles of making health data findable, accessible, interoperable and reusable (FAIR) that are currently proposed by different networks in Europe (51).

To make this possible, it will be essential to link initiatives, learning from best practices. Countries should learn from the lesson of the COVID-19 pandemic, making the secondary use of health data widespread, shared at minimal cost and highly sustainable. In this way, the convenience of cross-border collaboration would be demonstrated in practice by the immediate availability of data and indicators that cannot be easily gathered out of the boundaries of their own organizations.

Finally, some relevant limitations of our study are outlined below.

Firstly, the study was conducted on a limited sample of partners and collaborating institutions of the EUBIROD network. Although including key partners from 14 out of 27 current Member States of the EU, we cannot claim for our survey to represent a complete overview of the European context. Nevertheless, we included the majority of experiences operating on a permanent basis, which have been influential on the development of diabetes information systems both at national and international level, as shown by their inclusion also in other reviews.

Secondly, the review has been conducted in 2017. In a rapidly changing environment, this means that a substantial update will be needed soon, to ensure that the details we have provided here are still current. However, we have requested further information related to the number of patients relative to the last year available, showing that 10 out of 18 entities were still fully operational at least until 2019.

Thirdly, we did not directly verify the functionality and quality of indicators produced by the data sources, either by conducting interviews with local developers, or by running any statistical analysis on internal data. That is the scope of a new project, implementing the BIRO approach for the purpose of automating the overall analysis of a collaborating network (52). Further research will be required to evaluate procedures in each case.

In conclusion, accurate diabetes information is central to the strategy of quality of care and outcomes improvement in diabetes in many jurisdictions at national and sub-national level. Diabetes registries have demonstrated modern ways to organize, govern and deliver best information at regional, national and international level. However, the heterogeneous implementation across Europe still hampers the ability to provide accurate and comparable information on the achievement of common targets.

Best practices exist, but need to be shared more effectively to ensure that efforts are aligned. An area of particular interest is that of incentivised collaboration between central authorities and physician networks. The strengthening of international collaborations e.g. the EUBIROD network may accelerate the adoption of these approaches, but countries will need to invest more on health information systems that are interoperable and consistent with the expectations of people with diabetes, taking their perspectives into account. Future updates and extensions of the present survey will be needed to monitor progress in this rapidly evolving field.

## Author Contributions

The present paper is a collective product of the EUBIROD network, including main authors and other authors who contributed substantially to the conception and delivery of the survey. All authors provided an active contribution in the development of the survey instrument, data collection and production of the manuscript. FC and IS designed the survey questionnaire. IS implemented the online survey tool and extracted the data for analysis. FC has carried out the analysis and wrote the first draft of the manuscript. All authors have contributed to the writing and revision of the manuscript. All authors have read, and confirm that they meet, ICMJE criteria for authorship. All authors had full access to all of the data (including statistical reports and tables) in the study and can take responsibility for the integrity of the data and the accuracy of the data analysis. FC is the guarantor. All authors contributed to the article and approved the submitted version.

## Funding

The products presented in this study have been funded through granting provided by DG-SANCO in the EU project Bridge Health (GA 664691).

## Conflict of Interest

SL is the Director of the RCGP RSC, as part of his academic work. He has received grants through his institution from AstraZeneca, Eli Lilly, Novo, and Sanofi, for diabetes related research. SC is employed by My Digital Health. CI is employed by Serectrix snc. SP was employed by Telemedica Consulting.

The remaining authors declare that the research was conducted in the absence of any commercial or financial relationships that could be construed as a potential conflict of interest.

## Publisher’s Note

All claims expressed in this article are solely those of the authors and do not necessarily represent those of their affiliated organizations, or those of the publisher, the editors and the reviewers. Any product that may be evaluated in this article, or claim that may be made by its manufacturer, is not guaranteed or endorsed by the publisher.
